# The Association Between *STX1B* Polymorphisms and Treatment Response in Patients With Epilepsy

**DOI:** 10.3389/fphar.2021.701575

**Published:** 2021-07-09

**Authors:** Shitao Wang, Liang Zhou, Chenglu He, Dan Wang, Xuemei Cai, Yanying Yu, Liling Chen, Di Lu, Ligong Bian, Sunbing Du, Qian Wu, Yanbing Han

**Affiliations:** ^1^ Department of Neurology, The First Affiliated Hospital of Kunming Medical University, Kunming, China; ^2^ Department of Clinical Laboratory, The First Affiliated Hospital of Kunming Medical University, Kunming, China; ^3^ Biomedicine Engineering Research Center, Kunming Medical University, Kunming, China

**Keywords:** STX1B, epilepsy, polymorphism, association, treatment

## Abstract

**Background:** Epilepsy is a debilitating brain disease with complex inheritance and frequent treatment resistance. However, the role of *STX1B* single nucleotide polymorphisms (SNPs) in epilepsy treatment remains unknown.

**Objective:** This study aimed to explore the genetic association of *STX1B* SNPs with treatment response in patients with epilepsy in a Han Chinese population.

**Methods:** We first examined the associations between *STX1B* SNPs and epilepsy in 1000 Han Chinese and the associations between *STX1B* SNPs and drug-resistant epilepsy in 450 subjects. Expression quantitative trait loci analysis was then conducted using 16 drug-resistant epileptic brain tissue samples and results from the BrainCloud database (http://eqtl.brainseq.org).

**Results:** The allelic frequencies of rs140820592 were different between the epilepsy and control groups (*p* = 0.002) after Bonferroni correction. The rs140820592 was associated with significantly lower epilepsy risk among 1,000 subjects in the dominant model after adjusting for gender and age and Bonferroni correction (OR = 0.542, 95%CI = 0.358–0.819, *p* = 0.004). The rs140820592 also conferred significantly lower risk of drug-resistant epilepsy among 450 subjects using the same dominant model after adjusting for gender and age and Bonferroni correction (OR = 0.260, 95%CI = 0.103–0.653, *p* = 0.004). Expression quantitative trait loci analysis revealed that rs140820592 was associated with *STX1B* expression level in drug-resistant epileptic brain tissues (*p* = 0.012), and this result was further verified in the BrainCloud database (http://eqtl.brainseq.org) (*p* = 2.3214 × 10^–5^).

**Conclusion:** The *STX1B* rs140820592 may influence the risks of epilepsy and drug-resistant epilepsy by regulating *STX1B* expression in brain tissues.

## Introduction

Epilepsy is one of the most prevalent chronic neurological disorders worldwide, with an estimated globe prevalence > 0.5% (Shorvon, 1990; Fiest et al., 2017). The disease is characterized by episodes of hyper-synchronized neuronal activity leading to recurrent seizures. It is estimated that more than half of all epilepsy cases are associated with genetic factors (Pal et al., 2010). Mutations in many genes have been reported to cause epilepsy (Helbig et al., 2008; Poduri and Lowenstein, 2011; Ponnala et al., 2012; Abou El Ella et al., 2018; Butilă et al., 2018), and epilepsies associated with different mutations exhibit substantial heterogeneity in disease course, clinical manifestations, and treatment response (Wang et al., 2017), presenting significant challenges for diagnosis and management.

The *STX1B* gene (16p11) encodes Syntaxin 1B, a protein of the SNARE complex mediating calcium-dependent synaptic vesicle release (Südhof, 2013). Synaptic dysfunctions are associated with a myriad of neurological disorders, including epilepsy (Steinlein, 2004; Luscher and Isaac, 2009; van Spronsen and Hoogenraad, 2010; Lepeta et al., 2016; Torres et al., 2017). Recent studies suggest that *STX1B* is involved in epilepsy (Schubert et al., 2014; Wolking et al., 2019). However, the role of *STX1B* SNPs in epilepsy treatment remains unknown, so it is necessary to explore the genetic association of *STX1B* SNPs with treatment response in patients with epilepsy in a Han Chinese population.

In this study, we investigated the associations between seven *STX1B* tagging SNPs and treatment response in patients with epilepsy in Han Chinese, and then conducted brain expression quantitative trait loci (eQTL) analysis. The *STX1B* rs140820592 was associated with reduced risks for epilepsy and drug-resistant epilepsy, likely by regulating *STX1B* expression in brain tissues.

## Materials and Methods

### Subjects

A case‒control study was performed to investigate the associations between *STX1B* tagging SNPs and drug-resistant epilepsy. Clinical and demographic characteristics of the study cohort are summarized in Table 1. All blood samples were collected at the First Affiliated Hospital of Kunming Medical University, and stored at −80°C in the biobank of the First Affiliated Hospital of Kunming Medical University. All brain tissue samples were collected at the First Affiliated Hospital of Kunming Medical University and Xinqiao Hospital and stored at −80°C in the biobank of Kunming Medical University. All subjects included in this study were of Han Chinese ancestry. The epilepsy patients were diagnosed according to 2017 International League Against Epilepsy (ILAE) criteria, and drug-resistant epilepsy patients were diagnosed according to 2010 ILAE criteria. Carbamazepine, valproic acid, levetiracetam and Lamotrigine were prescribed to the epilepsy patients. In order to improve epilepsy care and research, the ILAE defined drug-resistant epilepsy as failure of adequate trials of two tolerated, appropriately chosen and used antiepileptic drug schedules (whether as monotherapies or in combination) to achieve sustained seizure freedom in 2010 (Kwan et al., 2010), and developed the new classification of seizures and epilepsy relevant to clinical practice in 2017 (Fisher et al., 2017; Scheffer et al., 2017). Symptomatic epilepsy was excluded through auxiliary examination and disease history review. The healthy controls had no individual or family history of epilepsy and were neurologically normal. All participates or legal representatives provided written informed consent in accordance with the tenets of the Declaration of Helsinki. All study protocols were approved by the Ethics Committee of the First Affiliated Hospital of Kunming Medical University (No.2020-L-40).

**TABLE 1:** Demographic characteristics of the patients and controls.

Characteristics	Epilepsy	Control	P^a^	DT	DE	p^b^
Males/Females	214/236	244/306	0.314	57/74	157/162	0.271
Age (Mean ± SD)	24.63 ± 16.221	25.77 ± 15.664	0.260	28.74 ± 17.162	22.95 ± 15.535	0.001

SD: standard deviation, DT: drug-resistant epilepsy, DE: drug-responsive epilepsy.

Difference in gender between cases and controls was analyzed using the χ2 test.

Difference in mean age was analyzed using the independent sample *t*-test.

a P-values were calculated between epilepsy and control.

b P-values were calculated between drug-resistant epilepsy and drug-responsive epilepsy.

### Selection and Genotyping of SNPs

We first identified all *STX1B* SNPs in the Chinese Han South (CHS) population recorded in the 1000 Genomes database (http://www.internationalgenome.org/), and then used Haploview software (Barrett et al., 2005) to pick seven tagging SNPs (*r* ^2^ > 0.8) with minor allele frequency > 5%. Basic information for seven *STX1B* SNPs is summarized in Table 2. Genomic DNA was extracted from human brain tissues using the Tissue DNA Kit (OMEGA, United States) and from peripheral blood using the Blood DNA Mini Kit (OMEGA, United States). SNP genotyping was conducted using the Bio-Rad CFX96 (BioRad, United States) platform. Primers for PCR were designed using Primer Premier V6.0 (Premier Biosoft Inc., United States). Details on PCR primers are provided in Supplementary Table 1.

**TABLE 2:** Basic information for seven *STX1B* SNPs.

SNP ID	Position	Location	Minor allele	MAF^a^	MAF^b^
rs4889606	Intron	31,011,183	A	0.1095	0.0995
rs8060857	3’ UTR	31,002,720	G	0.0667	0.079
rs12445568	Intron	31,004,812	C	0.0857	0.088
rs74474326	Intron	31,009,343	T	0.0857	0.1105
rs79086360	Intron	31,009,866	C	0.0571	0.0505
rs140820592	5’ UTR	3,1,021,880	T	0.081	0.0715
rs186050757	Intron	31,021,024	T	0.0571	0.034

SNP: Single nucleotide polymorphism, MAF: Minor allele frequency.

a MAF: Minor allele frequency in Han Chinese in 1000 Genomes project.

b MAF: Minor allele frequency in Han Chinese in the study population.

### Association Analysis of SNPs

SPSS V23.0 (IBM Corp, Armonk, NY) was used for all analyses. Haplotypes were constructed and analyzed using SHEsis software (http://analysis.bio-x.cn/myAnalysis. php). Hardy‒Weinberg equilibrium and allele frequencies of all SNPs were analyzed using the Chi-square (χ2) test or Fisher’s exact test. Difference in genotype frequencies of all SNPs between cases and controls was analyzed using binary logistic regression. In order to calculate the statistical power, type I error rate of 0.05, dominant mode, and 0.007 of baseline risk were assumed with QUANTO V1.2.4 (Written by John Morrison and W.James Gauderman at the University of Southern California), so the dominant model was also used to evaluate the associations between the genotypes of all SNPs and the risks of epilepsy and drug-resistant epilepsy.

### eQTL Analysis

To examine if the tagging SNPs associated with epilepsy and drug-resistant epilepsy were also associated with *STX1B* expression level in drug-resistant epileptic brain tissues, we conducted eQTL analysis using temporal lobe samples from 16 patients (nine males and seven females) with drug-resistant epilepsy. Genomic RNA was extracted using the Tissue RNA Kit (OMEGA, United States), and reverse transcribed into cDNA using the FastQuant cDNA kit (Tiangen, China). Quantitative PCR was conducted using SYBR^®^Green I (Vazyme, China) and the ABI QuantStudio six Flex™ (ABI, United States) analyzer. Primers were designed using Primer Premier V6.0 (Premier Biosoft Inc., United States) Relative expression levels were determined using the 2^−ΔΔCT^ method, and differences in *STX1B* mRNA expression between genotypes were analyzed by independent sample *t*-test using Graphpad Prism9.0 (www.graphpad- prism.cn). We also conducted eQTL analysis using results from the BrainCloud database (http://eqtl.brainseq.org) to validate eQTL findings from epileptic brain tissues. Details on qPCR primers are provided in Supplementary Table 2.

### Statistical Analysis

Data are expressed as number, frequency, or mean ± standard deviation (SD) as appropriate. Continuous variables were compared between groups by independent samples *t*-test and categorical variables by χ2 test, Fisher’s exact test and logistic regression analysis using SPSS V23.0 (IBM Corp, Armonk, NY) and Graphpad Prism9.0 (www.graphpad-prism.cn) A *p* < 0.05 (two-tailed) was considered significant. For Bonferroni correction, A *p* < 0.007 (0.05/7) was considered significant.

## Results

### Subject Characteristics

Neither average age nor gender ratio differed significantly between the epilepsy group and control group. However, average age is significantly older in the drug-resistant epilepsy group than drug-responsive epilepsy group. Subject information is summarized in Table 1.

### *STX1B* SNPs Associated Epilepsy Treatment Response in Han Chinese

To comprehensively evaluate the relationships between the seven selected SNPs and epilepsy treatment response in Han Chinese, we performed a case‒control study involving 1,000 individuals (450 cases, 550 healthy controls). All seven tagging SNPs were in Hardy‒Weinberg equilibrium among control participants (*p* > 0.05) (Table 3).

**TABLE 3:** Comparison of allele frequency distributions for seven tagging SNPs between cases and controls.

SNP ID	Alleles	P^a^	P^b^	P^c^	p^d^
rs4889606	G > A	0.199	0.359	0.769	0.948
rs8060857	A > G	0.881	0.423	0.799	1.000
rs12445568	T > C	0.409	0.139	0.998	0.947
rs74474326	C > T	0.523	0.576	1.000	1.000
rs79086360	T > C	0.479	0.097	1.000	0.882
rs140820592	G > T	0.002	0.028	0.712	1.000
rs186050757	C > T	0.552	0.035	1.000	0.825

SNP Single nucleotide polymorphism.

a p values were calculated using χ2 test for comparison of the allele distribution frequencies between epilepsy patients and healthy controls.

b P values were calculated using χ2 test for comparison of the allele distribution frequencies between drug-resistant epilepsy patients and drug-responsive epilepsy patients.

c P values were calculated using χ2 test or Fisher's exact test for Hardy-Weinberg equilibrium in healthy control.

d P values were calculated using χ2 test or Fisher's exact test for Hardy-Weinberg equilibrium in drug-responsive epilepsy patients.

In the Stage I study, we found a significant difference in rs140820592 allele distribution between the epilepsy group and the control group (*p* = 0.002) (Table 3), and the association remained significant after adjusting for age and gender and Bonferroni correction (OR = 0.542, 95%CI = 0.358–0.819, *p* = 0.004) in the dominant model (GT + TT vs. GG) (Table 4).

**TABLE 4:** Associations between *STX1B* SNP genotypes and epilepsy.

SNP ID	Genotype	Epilepsy N (%)	Control N (%)	OR (95% CI)	P
rs4889606 G > A	GG	372 (82.7)	436 (79.3)	1.000	–
	GA + AA	78 (17.3)	114 (20.7)	0.855 (0.595–1.227)	0.394
rs8060857A > G	AA	382 (84.9)	469 (85.3)	1.000	–
	AG + GG	68 (15.1)	81 (14.7)	1.178 (0.800–1.735)	0.406
rs12445568 T > C	TT	379 (84.2)	453 (82.4)	1.000	–
	TC + CC	71 (15.8)	97 (17.6)	0.964 (0.662–1.404)	0.849
rs74474326 C > T	CC	359 (79.8)	431 (78.4)	1.000	–
	CT + TT	91 (20.2)	119 (21.6)	1.014 (0.713–1.442)	0.939
rs79086360 T > C	TT	408 (90.7)	492 (89.5)	1.000	–
	TC + CC	42 (9.3)	58 (10.5)	0.970 (0.620–1.518)	0.894
rs140820592 G > T	GG	405 (90.0)	456 (82.9)	1.000	–
	GT + TT	45 (10.0)	94 (17.1)	0.542 (0.358–0.819)	0.004
rs186050757 C > T	CC	417 (92.7)	515 (93.6)	1.000	–
	CT + TT	33 (7.3)	35 (6.4)	1.354 (0.804–2.281)	0.254

SNP: Single nucleotide polymorphism, OR: Odds ratio, 95% CI: 95% confidence interval.

p-values calculated by logistic regression analysis with adjustment for gender and age.

In Stage II, we further explored the relationships between *STX1B* SNPs and drug-resistant epilepsy among 450 Han Chinese individuals (131 drug-resistant epilepsy patients, and 319 drug-responsive epilepsy patients), and again found this association remained after adjusting for age and gender and Bonferroni correction (OR = 0.260, 95%CI = 0.103–0.653, *p* = 0.004) in the dominant model (GT + TT vs. GG) (Table 5).

**TABLE 5:** Associations between *STX1B* SNP genotypes and drug-resistant epilepsy.

SNP ID	Genotype	Drug-resistant patients N (%)	Drug-responsive patients N (%)	OR (95% CI)	P
rs4889606 G > A	GG	112 (85.5)	260 (81.5)	1.000	–
	GA + AA	19 (14.5)	59 (18.5)	0.565 (0.303–1.055)	0.073
rs8060857A > G	AA	114 (87.0)	268 (84.0)	1.000	–
	AG + GG	17 (13.0)	51 (16.0)	0.577 (0.301–1.107)	0.098
rs12445568 T > C	TT	116 (88.5)	263 (82.4)	1.000	–
	TC + CC	15 (11.5)	56 (17.6)	0.452 (0.232–0.880)	0.019
rs74474326 C > T	CC	102 (77.9)	257 (80.6)	1.000	–
	CT + TT	29 (22.1)	62 (19.4)	0.963 (0.541–1.713)	0.897
rs79086360 T > C	TT	114 (87.0)	294 (92.2)	1.000	–
	TC + CC	17 (13.0)	25 (7.8)	1.408 (0.696–2.849)	0.341
rs140820592 G > T	GG	125 (95.4)	280 (87.8)	1.000	–
	GT + TT	6 (4.6)	39 (12.2)	0.260 (0.103–0.653)	0.004
rs186050757 C > T	CC	116 (88.5)	301 (94.4)	1.000	–
	CT + TT	15 (11.5)	18 (5.6)	1.718 (0.799–3.694)	0.166

SNP: Single nucleotide polymorphism, OR: Odds ratio, 95% CI: 95% confidence interval.

p-values calculated by logistic regression analysis with adjustment for gender and age.

**FIGURE 1:**
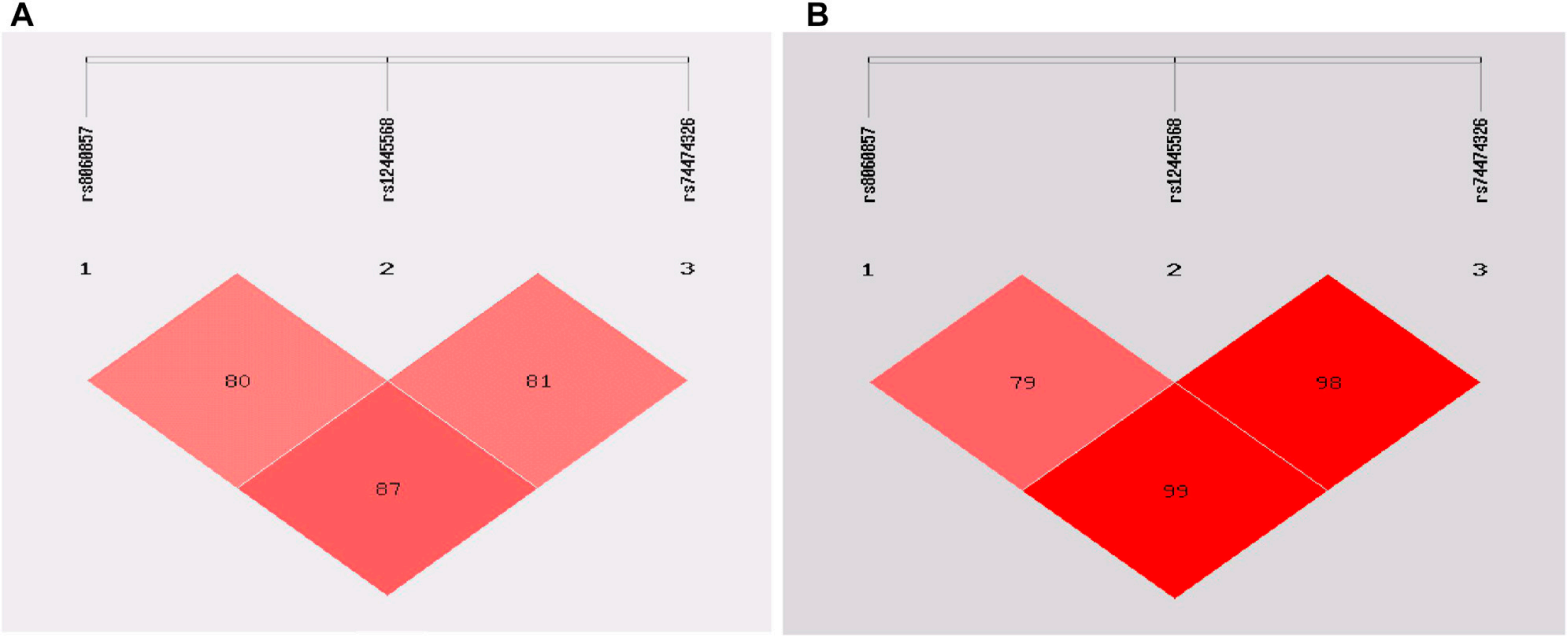
Linkage disequilibrium (LD) analysis of the *STX1B* SNPs in cases and controls: **(A)** in epilepsy patients and controls **(B)** in drug-resistant epilepsy patients and drug-responsive patients.

The rs8060857, rs12445568, and rs74474326 polymorphisms have strong linkage disequilibrium for both epilepsy vs. control groups and drug-resistant epilepsy vs. drug-responsive epilepsy groups (Figure 1). There were no significant difference in the distributions of haplotypes A-T-C, A-T-T, and G-C-C between epilepsy and control groups (*p* = 0.812, *p* = 0.726, and *p* = 0.938, respectively) (Table 6 **)** or between drug-resistant epilepsy vs. drug-responsive epilepsy groups (*p* = 0.936, *p* = 0.636, and *p* = 0.478, respectively) (Table 7).

**TABLE 6:** Association between Haplotypes of *STX1B* SNPs and epilepsy risk.

Haplotype	Epilepsy	Control	X^2^	p	OR (95% CI)
A-T-C	718.04 (0.798)	863.76 (0.785)	0.057	0.812	1.029 (0.814–1.301)
A-T-T	94.72 (0.105)	119.85 (0.109)	0.123	0.726	0.950 (0.714–1.264)
G-C-C	58.73 (0.065)	70.07 (0.064)	0.006	0.938	1.014 (0.708–1.452)

OR Odds ratio, 95% CI 95% confidence interval.

The haplotypes are combined with *STX1B* rs8060857-rs12445568-rs74474326. Haplotypes (frequency < 3%) in both groups have been ignored.

**TABLE 7:** Association between Haplotypes of *STX1B* SNPs and drug-resistant epilepsy risk.

Haplotype	Drug-resistant epilepsy	Drug-responsive epilepsy	X^2^	p	OR (95% CI)
A-T-C	213.00 (0.813)	505.13 (0.792)	0.007	0.936	1.016 (0.693–1.490)
A-T-T	29.99 (0.114)	64.62 (0.101)	0.224	0.636	1.117 (0.706–1.769)
G-C-C	14.98 (0.057)	43.72 (0.069)	0.504	0.478	0.803 (0.438–1.472)

OR Odds ratio, 95% CI 95% confidence interval.

The haplotypes are combined with *STX1B* rs8060857-rs12445568-rs74474326. Haplotypes (frequency < 3%) in both groups have been ignored.

### eQTL Analysis

**FIGURE 2:**
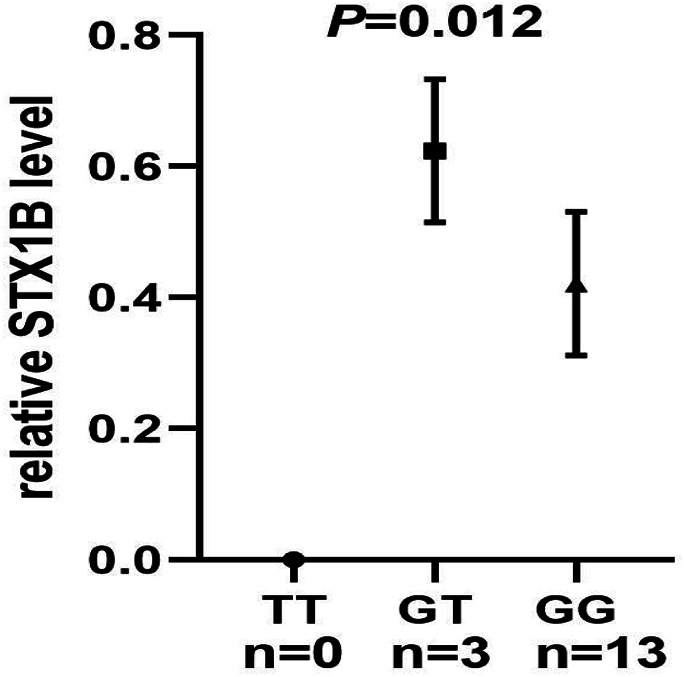
The rs140820592 is an eQTL in temporal lobe tissue of drug-resistant epilepsy patients. The carriers of the T allele exhibited upregulated STX1B gene expression.

**FIGURE 3:**
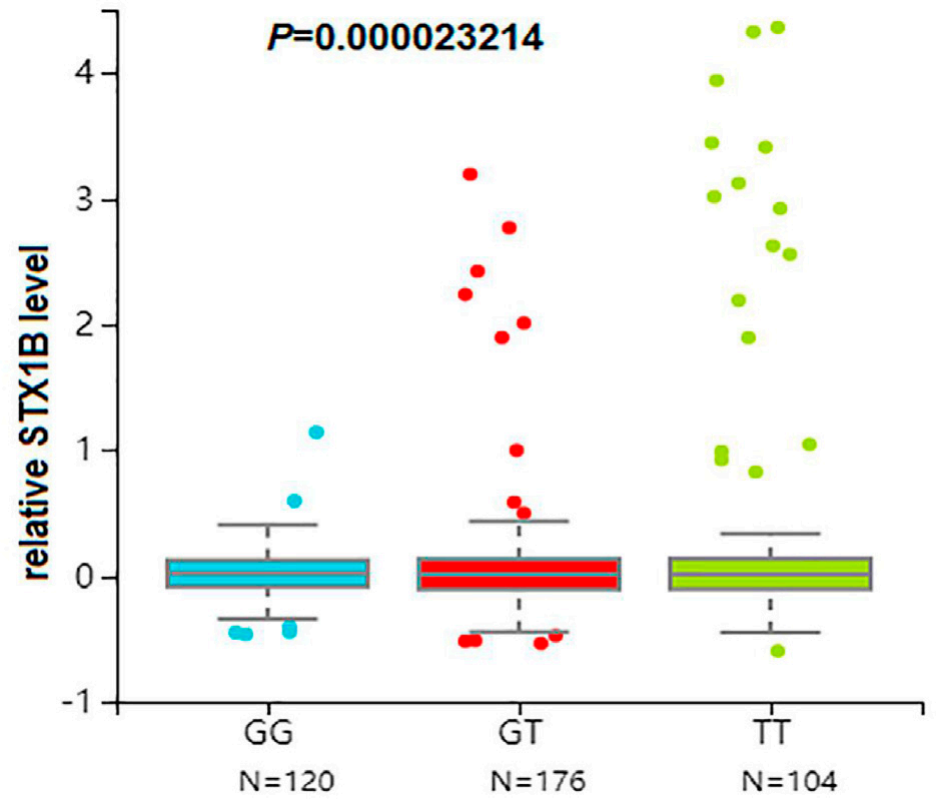
The rs140820592 is an eQTL in the dorsolateral prefrontal cortex. Data were retrieved from the brain tissue database Braincloud (http://eqtl.brainseq.org).

We then conducted eQTL analysis based on brain tissue samples from 16 epilepsy patients with drug-resistant epilepsy and found that T allele carriers of rs140820592 exhibited higher *STX1B* expression than GG carriers (*p* = 0.012) (Figure 2), a finding consistent with records from the BrainCloud database (http://eqtl.brainseq.org) (Figure 3).

## Discussion

Syntaxin 1B together with its binding partner Syntaxin binding protein 1 (Hata et al., 1993; Ogawa et al., 1998) is a critical component of the minimal presynaptic transmitter release machinery (Jahn and Sheller, 2006; Rizo and Sudhof, 2012). Here we showed that rs140820592 in the Syntaxin 1B-coding gene *STX1B* was significantly associated with the risks of epilepsy (drug-resistant epilepsy + drug-responsive epilepsy) and drug-resistant epilepsy. This is a study combining genetic association and eQTL analyses in brain tissues and blood samples to comprehensively evaluate the relationship between the *STX1B* gene and epilepsy treatment.

Among the seven tagging SNPs, the SNP rs140820592 was significantly associated with epilepsy. Specifically, risk of epilepsy was lower in rs140820592 GT + TT genotype carriers in the dominant model after adjusting for gender and age, and significance was maintained after Bonferroni correction (Table 4). Furthermore, eQTL analysis showed that the T allele carriers of rs140820592 was associated with increased *STX1B* expression in drug-resistant epileptic brain tissues (Figure 1), suggesting rs140820592 is a functional SNP.

In addition to epilepsy (drug-resistant epilepsy + drug-responsive epilepsy), the rs140820592 GT + TT genotype decreased the risk of drug-resistant epilepsy in the dominant model after adjusting for gender and age, and significance remained after Bonferroni correction (Table 5). To our knowledge, there are no other reports on the relationship between *STX1B* SNPs and drug-resistant epilepsy. In summary, rs140820592 may decrease the risks of epilepsy and drug-resistant epilepsy by regulating *STX1B* gene expression in human brain tissues. However, further larger sample studies are required for confirmation.

Haplotype analysis showed a strong LD for rs8060857, rs12445568, and rs74474326 (D’ = 0.79–0.99) in this study. However, the frequencies of these haplotypes, including A-T-C, A-T-T and G-C-C, did not differ between case and control groups for either epilepsy or drug-resistant epilepsy (Table 6 and Table 7), at least in part because these SNPs were not associated with epilepsy or drug-resistant epilepsy in this study cohort. We did not find a strong LD between rs140820592 and other tagging SNPs, suggesting that rs140820592 may be an independent factor reducing the risks of epilepsy and drug-resistant epilepsy in Han Chinese.

Syntaxin 1B is mainly expressed in neurons, and has similar physiological functions to STX1A (Inoue et al., 1992; Kushima et al., 1997). The *STX1B* gene is highly expressed in temporal lobe according to BioGPS (http://biogps.org), so this region was chosen for eQTL analysis. Indeed, this analysis showed that rs140820592 is an eQTL in drug-resistant epileptic brain tissues. As reported in previous studies, disease-associated SNPs often influence the risk of disease by regulating gene expression (Nicolae et al., 2010; Maurano et al., 2012; Ward and Kellis, 2012; Bi et al., 2018; Guo et al., 2018; Li et al., 2018; Wang et al., 2021), and this result further verified the association between rs140820592 and epilepsy treatment response. These findings may aid in the development of new diagnostic methods and therapeutic targets for epilepsy.

Genetic factors have been found to affect drug response in many studies (Franco and Perucca, 2015; Balestrini and Sisodiya. 2018). Understanding the associations between *STX1B* SNPs and drug response will aid in translation of genetic findings to clinical treatment. We found that the *STX1B* rs140820592 was significantly associated with the risk of drug-resistant epilepsy at the genetic and expressional levels, suggesting a role of *STX1B* in epilepsy treatment*.* STX1B plays an important role in the regulation of synaptic vesicle exocytosis, including release of glutamatergic and GABAergic synaptic transmission (Mishima et al., 2014). Furthermore, some important neurotransmitters, such as glutamate and GABA have been found to be involved in epilepsy and drug treatment (Teichgräber et al., 2009; Ngomba and van Luijtelaar, 2018; Chun et al., 2019; Alcoreza et al., 2021). Therefore, STX1B may be involved in epilepsy and drug responsiveness by regulating synaptic vesicle exocytosis.

However, our study in Han Chinese failed to replicate rs140820592 minor allele (T) as a increased risk for epilepsy in the United Kingdom biobank database. The opposite direction of the effect can be explained as follows: First, the difference in the distribution of alleles among different populations should be considered. Second, study subjects for different types of epilepsy may also draw inconsistent conclusions.

Although we have provided important evidence that STX1B gene is involved in the development of epilepsy and drug-resistant epilepsy at the genetic and expressional levels in Han Chinese, some limitations of this study should also be noted: First, our study only included the Han population, and the sample size was relatively small (We estimated that our study had 80% power to detect significant SNPs of MAF = 0.034 with relative risk >1.75.), so a larger sample study including different ethnic groups should be conducted to further verify our findings. Second, we performed functional analysis only in human brain tissues, and further experiments are necessary. Third, in this study, only STX1B gene was included, and other important epilepsy genes were not included in our study.

## Conclusion

We provide multiple lines of evidence that the *STX1B* rs140820592 may decrease the risks of epilepsy (drug-resistant epilepsy + drug-responsive epilepsy) and drug-resistant epilepsy by regulating *STX1B* expression. STX1B gene may be a new therapeutic target for epilepsy. However, It is necessary to further verify our conclusions through studies with large samples in the future.

## Data Availability

The original contributions presented in the study are included in the article/Supplementary Material, further inquiries can be directed to the corresponding author.
